# Pathological fractures of the proximal femur due to solitary bone cyst: classification, methods of treatment

**Published:** 2015

**Authors:** A Miu

**Affiliations:** * “Dr. Carol Davila” Central Military University Emergency Hospital, Bucharest, Romania

**Keywords:** child, solitary bone cyst, femur fracture, minimally invasive treatment

## Abstract

Fractures are a very important issue in a child’s orthopedic pathology. Neglected a good amount of time, being considered “not too serious”, or “rare”, having better and faster healing methods and not leaving sequels, like in the case of adults, a child’s fractures remain an important chapter of traumatology in general.

Because of the raising prevalence of child osteoarticular traumas, as well as new less invasive treatment methods, this theme is always to date. The paper analyzes particular cases of bone fractures that appeared due to minor traumas, on bones with a high brittleness, localized especially on the long bones. Although these fractures on a pathological bone can be seen at all levels of the human skeleton, this paper focuses on fractures located in the proximal third part of the femur.

A group of children admitted in the Pediatric Orthopedic Department of “M.S. Curie” Hospital-Bucharest with this diagnostic, were analyzed between 2009 and 2013.

## Aim of the study 

Proximal femoral fractures are extremely rare on children, the clinical symptomatology is spectacular, the treatment methods are strictly particularized to the specific anatomy of the child. The chosen treatment method must be as invasive as possible, it must respect the particular anatomy of the region and it must prevent recurrence (given the superimposed pathology - the solitary bone cyst), and sequels. The goal of this study was to review an important clinical material, correlation with the appearance and size of cyst, individualized therapeutical approach for each and every case, “technical” difficulties that may occur – in correlation with osteosynthesis or the unlikely evolution of some particular cases, and last but not least, complications that may appear and their solution.

## Materials and method

The study was composed of 27 patients, with the average age of 11 years, admitted in “M.S. Curie” Hospital’s Pediatric Ortopaedic Clinic in Bucharest, during 2009 and 2013 (retrospective and prospective), with the following diagnosis: Pathological fracture (solitary bone cyst) of the proximal femur. Every patient in this study has suffered a surgical treatment, depending on the presentation of the disease. Both the treatment methods and the post operatory evolution were studied. The classification of the fractures was made according to the Delbet-Colona and AO Trauma classifications. The diagnosis was set based on the radiological examination, CT, as well as the histopathological examination, taken after the intraoperatory biopsy. 

## Results

Out of the 27 patients operated in the clinic, for 17 the method was intramedullary nailing with 2 TEN nails and filling with substitute materials, for one of the patients- PFNA and filling with Arex Bone was used, 8 patients had a minimum invasive resection-biopsy and filling with replacement material and a particular case, relapsed, needed osteosynthesis with an external fixator. The patients were observed post-intervention at 3, 6, 12 months, clinical and radiological. The radiological aspect showed the healing of the fracture, incorporation of the replacement material, maintenance of the trochanteric angle at a normal scale, lack of signs of avascular necrosis. The osteosynthesis material was extracted after 6-12 months. The evolution was favorable in most of the cases, only one recurrence was observed in this study. 

Child fractures are different from adult fractures; children’s bones are more elastic, the periosteum is thicker, usually, remaining intact (“green stick” fracture); that is why displaced fractures are rarer. At the long bones, we find the epiphysis and growth cartilages that must be preserved during therapeutic maneuvers to avoid growth disturbance and occurrence of angular deformities.

The determined causes in the appearance of bone fractures are traumas, mechanisms of torsion, pressure, traction. In their appearance, favorable aspects intervene: general (M. Lobstein, rickets, osteomalacia) and local (solitary bone cyst, osteosarcoma, osteomyelitis, etc.). Pathological fractures appear due to minor traumas/ injuries, on bones with a modified histological structure.

The paper studied only the group of pathological fractures that is favored by the presence of a solitary bone cyst located in the proximal third of the femur. These represent only 1% of the child’s femur fractures, being mainly characteristic to elders [**9**,**12**].

According to location, fractures of the proximal femur can be: 

a) According to Delbet-Colonna classification:

• type I - trans epiphyseal

• type II - trans cervical

• type III – cervicotrochanteric

• type IV – subtrochanteric

**Fig. 1:**
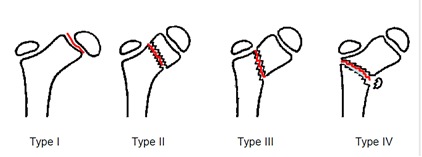
Classification of proximal thigh bone fractures (Delbet-Colonna)

b) according to AO Trauma classification

• type I – mid-cervical

• type II – basicervical

• type III – trans-trochanteric

**Fig. 2:**
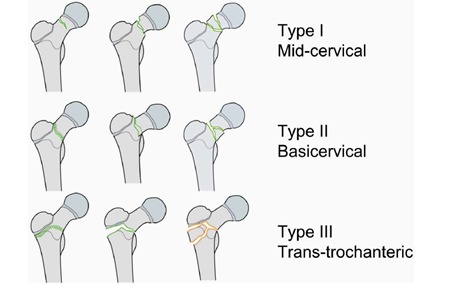
AO Trauma classification of child proximal femur fractures

This study addressed the proximal femur fractures on a preexistent lesion: essential bone cyst. 

**Definition:** the essential bone cyst is a benign bone lesion, which consists of a singular cavity, lined by a membrane, filled with yellowish serous fluid, present in the metaphysis of the long bones of the child and adolescent. It is a disease of the growing skeleton, which appears at first childhood and adolescence [**1**,**2**].

It mainly affects the male subjects (2:1), representing 3% of the child’s benign tumors [**1**,**2**,**7**,**14**,**15**]. The main location is at the proximal humerus (50%), followed by the proximal third of the femur (20%); the evolution potential is determined by the distance to the growth cartilage. This way, the lesions situated adjacent to the growth cartilage are considered active with a bad outcome, while lesions further away are considered dormant. This is determined in the choice of therapeutical attitude, the decision of starting a surgical treatment being associated with symptomatology, location and predictable outcome of the disease [**2**-**7**,**9**-**12**,**14**,**16**,**17**].

**Subjective criteria** that determine the opportunity of surgical treatment: 

• pain during moderate physical exercise;

• partial functional impairment (important aspect in the case of athletic and hyperactive adolescents)

**Objective criteria** are associated with serial radiological aspects: 

• thinning of cortical bone over time with the increased risk of fractures

• increase of cystic index over repeated radiological examinations. According to A. Kaelin & McEwen, the use of cystic index imposes a surgical intervention. They recommend a clinical observation in the case of humerus location with CI<4 and femoral with CI<3,5 [**13**];

**Fig. 3:**
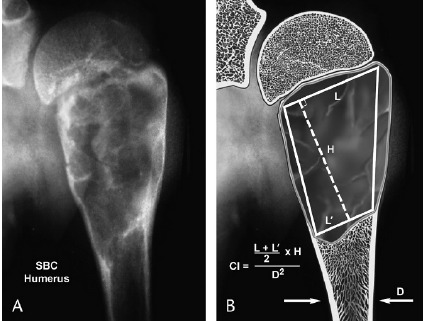
Cystic index calculation (A. Kaelin)

• juxta metaphyseal active lesions

• localization on the lower limb

• growth disorders with important bone deformities [**18**].

• iterative fractures

• age of the patient; the younger he/ she is, the more frequent are the relapses 

In these cases, the accuracy of the applied treatment is a mandatory condition. Sequels appeared after a bad treatment or wrong therapeutical indications are hard or even impossible to solve. It must be taken into account that the patients are growing children, with a high remodeling potential, but the model on which the skeleton is going to shape, needs to be correct from an anatomical and functional point of view.

Predictable sequels in the evolution of pathological proximal femoral fractures (solitary bone cyst) are: 

• avascular necrosis of the femoral head, which appears in most cases of transphyseal, transcervical and a large percent of the cervicotrochanteric fractures (28%) [**9**] 

• pseudarthrosis (6,5-13%

• bad consolidation with secondary coxa vara or premature closing of the growth plate (20-30%) [**18**].

• a particular case from the medical literature is the case of a spontaneous epiphyseal injury of the femoral neck in a 8-year-old boy, seen before for a pathological fracture, caused by a simple bone cyst which developed after 8 months a slipped capital femoral epiphysis. The physiopathology model proposed by the authors consisted of the hypothesis that the cystic lesion determined the epiphyseal injury followed by severe growth disturbance and varus deformity of the femoral head [**9**].

## Method

The retrospective and prospective study included 27 patients admitted in the Pediatric Orthopedics Clinic of “M.S. Curie” Children Hospital during 2008 and 2013, diagnosed with pathological fracture (s.b.c.) of the proximal femur. All the patients were informed and we had the parents’ permission of including them in the study. The study consisted of the treatment methods particularized to each case, as well as the clinical and radiological evolution of the patient. 

All the patients were previously diagnosed with solitary bone cyst located on the proximal femur. The diagnose was made by radiological examination and eventually computerized tomography (complex cases). During the surgery, we also took biopsies of the cystic lesions, which confirmed the initial diagnosis. 

Out of 41 children admitted in this period, with pathological fracture of the femur due to solitary bone cyst, 27 patients had the lesion in the proximal femur and 24 in the distal femur. Out of the 27 patients, 20 were boys. The localization in the proximal femur was 4-pertrohanterian, 5-intertrochanteric, and 18 subtrochanteric. The post-op evolution of every patient was favorable; we did radiological check-ups and 3, 6 and 12 months post-op. In one particular case, the tumoral lesion relapsed and a subsequent operation was necessary after 1 year.

**Table 1:** Classification of patients according to age, sex, number of surgeries undergone, treatment, and localization

NO	TREATMENT	AGE	LOCALIZATION	NO. OF SURGERIES	SEX	FOLLOW-UP
1	TEN+Arexbone	12	SUBTROCHANTERIC	1	M	6 MO
2	RESECTION-BIOPSY	10	SUBTROCHANTERIC	1	M	3 MO
3	TEN+Arexbone	13	SUBTROCHANTERIC	1	M	3 MO
4	BIOPSY	12	PERTRCOHAN-TERIC	1	F	3 MO
5	CURETTAGE-BIOPSY-EXCISION	10	PERTRCOHAN-TERIC	1	F	3 MO
6	RESENCTION+ILIAC AUTOGRAFT+EXTERNAL FIXING	12	SUBTROCHANTERIC	2	M	24 MO
7	RESECTION-BIOPSY	11	SUBTROCHANTERIC	1	M	12 MO
8	RESECTION-BIOPSY	10	INTERTROCHANTERIC	1	M	12 MO
9	TEN+CAUTERIZATION+ ArexBone SEALS	8	SUBTROCHANTERIC	1	M	3 MO
10	TEN+ArexBone	13	SUBTROCHANTERIC	2	M	3 MO
11	TEN+CAUTERIZATION+ArexBone	8	SUBTROCHANTERIC	1	M	3 MO
12	RESECTION-BIOPSY	11	SUBTROCHANTERIC	1	M	12 MO
13	BIOPSY	12	SUBTROCHANTERIC	1	M	12 MO
14	PFN+ArexBone+Alphagraft	15	INTERTROCHANTERIC	2	M	12 MO
15	BIOPSY-CAUTERIZATION+ProDense	8	INTERTROCHANTERIC	1	F	12 MO
16	ORTHOPEDIC REDUCTION +TEN+BIOPSY+PRODENSE	10	PERTROCHAN-TERIC	1	M	12 MO
17	TEN+BIOPSY+CAUTERIZATION	11	INTERTROCHANTERIC + MEMBERS DISPARITY	1	F	12 MO
18	RESECTION-BIOPSY+COSTAL ALLOGRAFTS SEAL	10	SUBTROCHAN-TERIC	1	M	12 MO
19	BIOPSY-CURETTAGE-CAUTERIZATION-ALLOMATRIX	6	SUBTROCHAN-TERIC	1	M	12 MO
20	BIOPSY-CURETTAGE+TEN	9	SUBTROCHAN-TERIC	1	M	12 MO
21	BIOPSY-CURETTAGE+TEN	5	PERTROCHAN-TERIC	1	M	12 MO
22	RESECTION -BIOPSY+TEN	16	SUBTROCHAN-TERIC	1	F	12 MO
23	EXT TRACTION +BIOPSY+TEN	12	SUBTROCHAN-TERIC	1	F	12 MO
24	RESECTION -BIOPSY+TEN	10	SUBTROCHAN-TERIC	1	M	20 MO
25	RESECTION -BIOPSY+TEN	9	INTERTROCHANTERIC	1	F	24 MO
26	RESECTION -BIOPSY+TEN	11	SUBTRCOHAN-TERIC	1	M	24 MO
27	RESECTION -BIOPSY-CAUTERIZATION+TEN	13	SUBTROCHAN-TERIC	1	M	24 MO

The therapeutical options in the treatment of the solitary bone cyst are multiple and individualized. The benign nature of the lesion allows the choice of the optimal surgical procedure. Because the majority of the cases admitted in the clinic are complicated, relapsed, the elected treatment is the surgical one.

The objectives of the surgical treatment are: 

• reduction of the fracture with anatomical alignment 

• preserving the epiphyseal presupposes the avoiding of complications that can appear during the development and growth

• restoration of the cervico-diaphyseal angle in order to avoid the coxa vara deformation

**Fig. 4:**
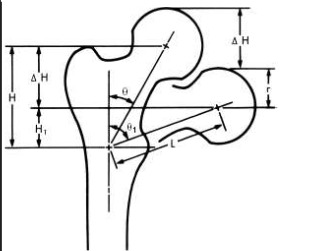
Cervico-diaphyseal angle in coxa vara deformation

• filling the bone defect with substitute material, so as to achieve the normal bone structure

Restoring the bone integrity can be accomplished by: 

a) orthopaedic reduction and skeletal traction, followed by a casting to maintain the reduction. This method is rarely used, being indicated only in the case of cystic lesions situated near the metaphysis. In this situation, the osteosynthetic material could damage the growth plate, followed by disturbances of a bone’s growth and development. Subsequent operations for the solitary bone cyst will be necessary after the healing of the fracture by various procedures, including curettage and packing with bone grafts.

b) open reduction with internal fixation eventually followed by filling of the bone cavity.

In the group of the patients of the study, various surgical interventions such as the following were performed:

• 8 patients were treated with elastic intramedullary nailing and filling with: ProDense (1 patient), ArexBone (5 patients), allograft (1 rib and 1 ilium) 

• 9 patients with intramedullary nailing, without filling

• 10 patients received a minimally invasive surgery, consisting of percutaneous treatment, curettage, cauterization, biopsy-resection and filling with bone graft (allograft, autograft and alloplastic materials) 

• 1 patient needed proximal femoral nail and filling with ArexBone and Alpha graft

• in the subsequent operation, the elastic intramedullary method and filling with Arex Bone was chosen in 1 patient who was initially operated with external fixator and iliac graft 

**Fig. 5:**
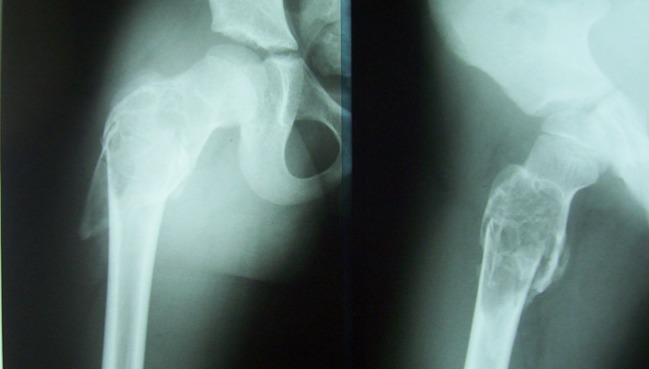
Before surgery

**Fig. 6:**
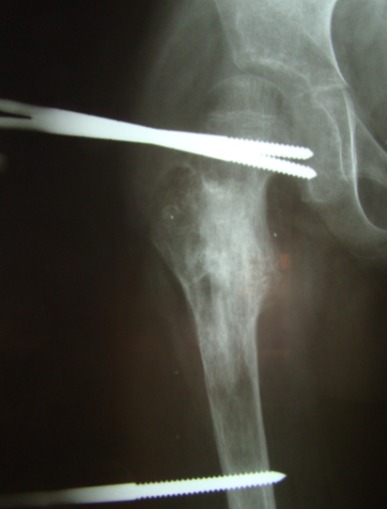
Postoperative aspect

**Fig. 7:**
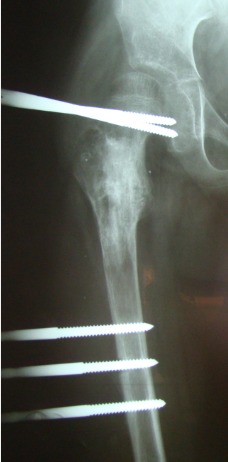
Pathological fracture 6mo postoperatory

**Fig. 8:**
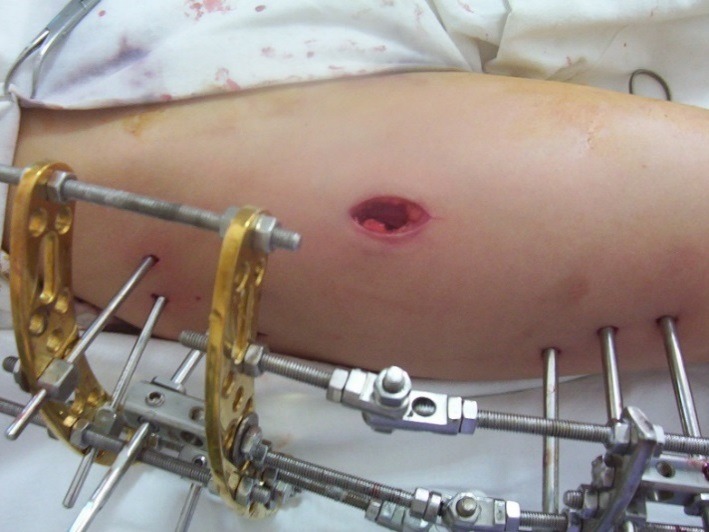
Intraoperative aspect-minimal skin incision

**Surgical techniques**

Operative treatment with elastic nails (TEN or Ender) [**3**-**5**,**8**]

• It is a minimally invasive treatment that requires general anesthesia. It begins with a small incision at the supracondylar region of the femur after the fluoroscopic visualization of the growth plate. The elastic nails are inserted retrograde through the femoral condyle. 

• The proper nail entry point is 2-3 proximal to the distal femoral physis, just below the metaphyseal-diaphyseal junction. A longitudinal skin incision is made on the lateral side; the incision is deepened with a hemostat to spread down to the bone. A drill is used to open the femoral cortex (the size of the drill beat should be larger than the size of the nail). This drilled hole will be the entry point for the elastic nails. 

• Under fluoroscopic imaging, after reducing the fracture, the bent nails are introduced across the fracture side. The nail inserted through the lateral femoral cortex should end laterally near the apophysis of the greater trochanter. The nail inserted through the medial distal femur should end at the lesser trochanter or femoral neck. For this kind of fracture, the nails can be introduced one anterograde through the greater trochanter and the other retrograde by lateral approach. Meanwhile, the cystic lesion can be treated by puncture-aspiration, fluoroscopically guided. This rupture of the lining and the septations within the cyst using the trocar; the cyst fluid is sent for pathologic analysis. Curettage can also be performed together with the cauterization of the cystic walls. In larger defects, the cavity will be filled with the allogenic or alloplastic substitute materials through minimal approach. The boney surface is exposed due to the fracture, the curettage is available, and the cauterization prevents the relapses. The osteosynthesis material insures stability and stimulates the osteogenesis of the osteolytic lesion. 

• Antibiotic prophylaxis is performed post-operatively. Usually, casting is not used – only in smaller children, because they are not compliant to the treatment. Early mobilization at seven days post-op is required. The weight baring is permitted at 6 weeks after the X-ray check-up. Follow-ups are performed at 3, 6, and 12 months – when the nails are extracted - post-op. The physical activity is allowed after the radiological healing of the lesion. As therapeutical results of the patients treated in the clinic, the results were satisfactory with a complete healing at 9-12 months. The radiological aspect emphasized the shrinking of the tumoral lesion, the thickening of the bone cortex, the growing of the density of the bone defect. 

• The advantages of this technique are the following:

- the minimally invasive procedure

- preserving the growth plate

- post-operative scars are small

- avoids damaging the femoral head circulation

- short admission period

- early mobilization

- keeping the fractural hematoma determines early and good consolidation 

• The disadvantages are the following: 

- the necessity of the C-Arm fluoroscope intra-operatory

- the high cost of the elastic nails 

**Fig. 9:**
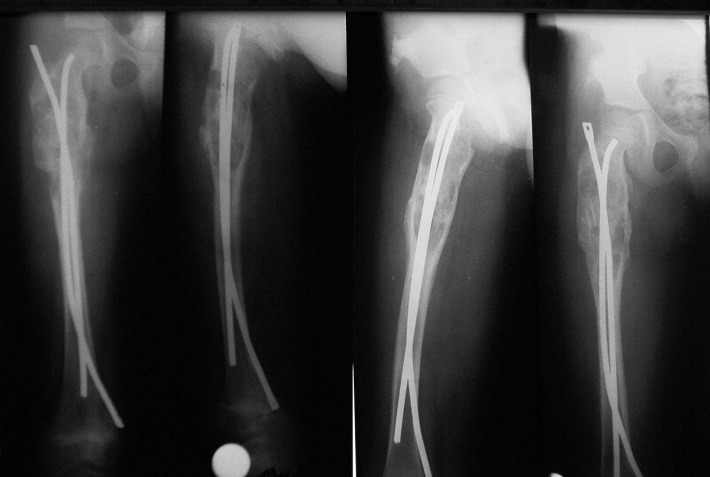
Solitary bone cyst of the proximal femur. Pathological fracture. Osteosynthesis with Ender nail - postoperatory aspect and 6 months follow-up

**Fig. 10:**
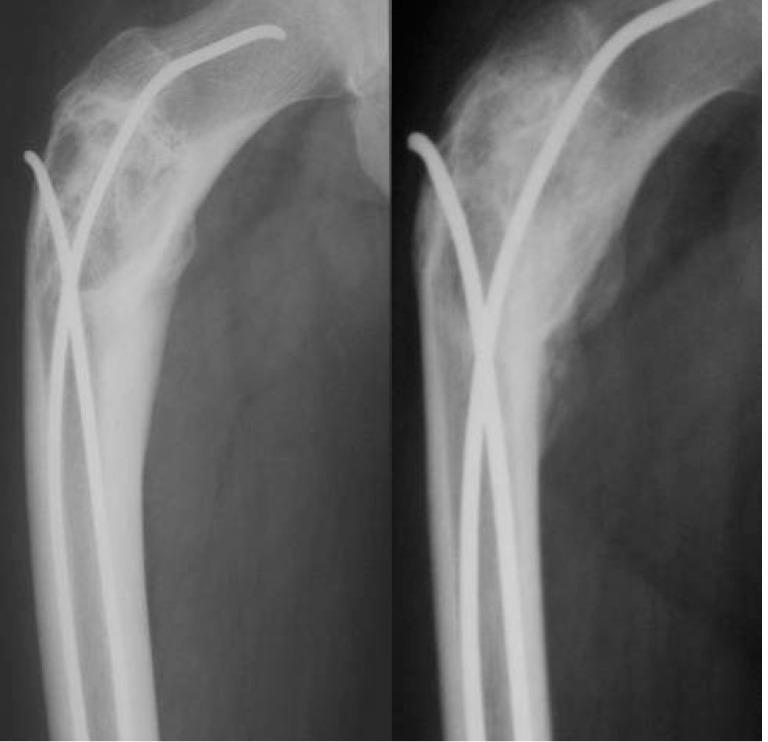
Pathological fracture of the proximal femur - postoperatory aspect and 3 months follow-up after TEN insertion

**The operative treatment of using a proximal femoral nail**

In 1958, AO Trauma postulated four basic principles that became directions for the internal fixation generally and intra-medullary osteosynthesis in particular. These are the following: 

- perfect anatomical reduction, followed by insertion of a guide pin

- stable fixation

- the preserving of the blood supply (minimal reaming of the medullar canal) 

- early and active mobilization 

In this patients’ series, we had only one case that needed the insertion of a PFN and filling with ArexBone and Alpha Graft. The patient was a 15-year-old boy, who presented four years before a proximal femoral fracture due to solitary bone cyst (histopathological exam proven). Initially, the treatment was conservative, with immobilization in spica cast until the healing of the fracture. After a 4-year period of health, the athletic child fell and suffered the same lesion after a football game. The surgical treatment was required (in another clinic of pediatric orthopedics, and after the fracture reduction, osteosynthesis was performed with 3 Kirschner nails introduced through the femoral neck. After 3 months, the nails were extracted and it was noticed that the cervico-diaphyseal angle was enlarged. It was decided that the child should be transferred in our clinic for further treatments. 

After a pre-operative evaluation, the patient was taken to the operating room, where a fracture table and a fluoroscopy were available. General anesthesia was preferable. After a large skin incision, from the superior iliac spine through the proximal third of the thigh, the muscle of the thigh was spread through the femoral neck, where the fracture sight lied. Skeletal traction was performed pre-operatively, so the fracture was aligned. After exposing the cystic lesion, cystic curettage (harvesting bone material for biopsy), cauterization of the cystic walls, disruption of the septations, were performed. As osteosynthesis material, PFNA (proximal femoral nail antirotator) was chosen for the establishment of the particular anatomy of the region. The cavity was filled with ArexBone and Alpha Graft. For the patient’s safety, an antirotation boot was put for 14 days. The 3 months follow up showed an excellent clinic and radiological evolution.

**Fig. 11:**
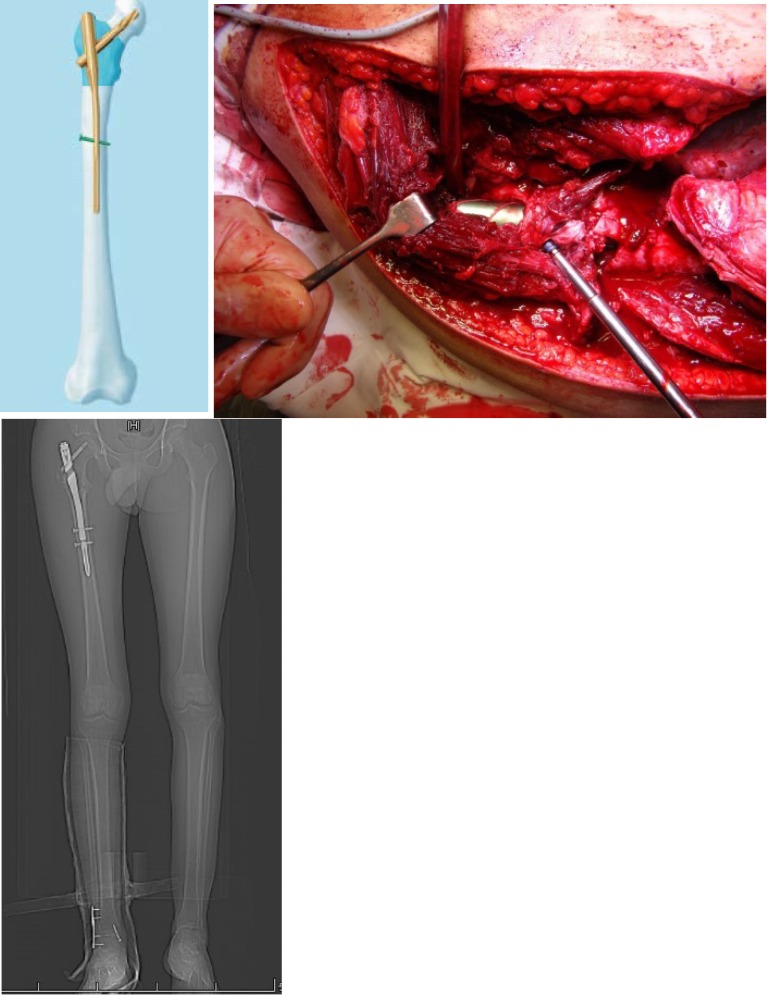
PFNA – intraoperative image - after surgery aspect

**Minimally invasive technique – without osteosynthesis [**2**,**3**,**6**,**8**-**10**,**15**,**16**]**

Mainly, a small incision is made after fluoroscopic guidance, followed by an optic curettage of the lesion, the cauterization of the cystic wall and the filling with bone graft. In this study group, 10 patients were treated this way. The radiological follow-up was performed at 3, 6, 12 months with good results. The healing of the fracture was noticed together with the filling of the bone defect.

**Fig. 12:**
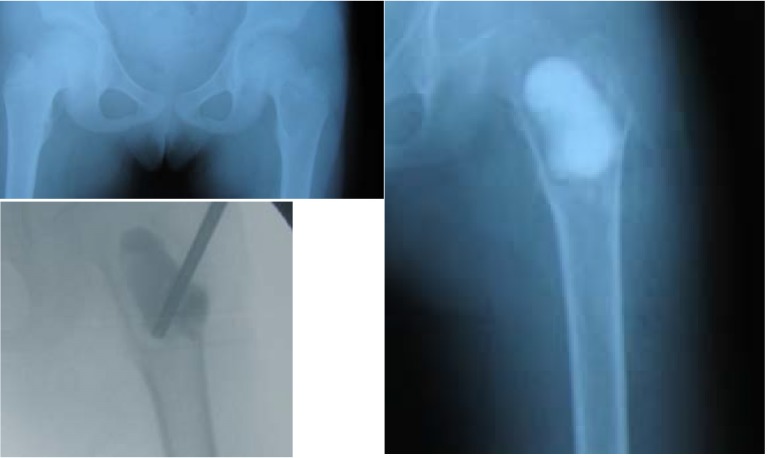
Solitary bone cyst of the proximal femur filled with Pro-Dense

**Fig. 13:**
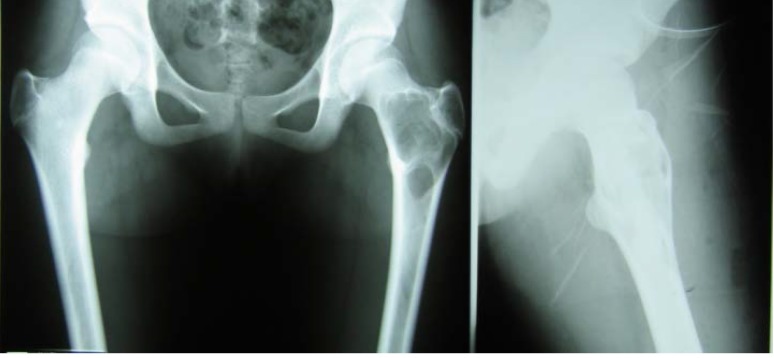
Solitary bone cyst filled with autologous bone graft: initial aspect and 6 months follow-up

## Results

Altogether, 27 patients diagnosed with pathological proximal femoral fractures in solitary bone cyst were studied during 2008 and 2013. All the patients suffered surgical treatment of various complexity. 20 patients were male, the average age was 11 years old. 

The location of the fracture sight: 

- 4 pertrochanteric

- 5 intertrochanteric

- 18 subtrochanteric

Regardless of the surgical procedure, there were not significant differences in the outcomes. The clinical and radiological follow-ups were satisfactory, with complete healing of the fracture, thickening of the bone cortex, filling of the boney defect, rising of the bone density inside the cyst. Relapse existed just in one case, which required a further intervention (followed by healing). In this time, we did not encounter any sequels (growth arrest, the absence of deformation, absence of avascular necrosis).

## Conclusion

The solitary bone cyst continues to raise many therapeutical challenges. The aim of the clinical studies is the less invasive surgeries, less post-operative complications, nevertheless the social aspect, the early recovery of the children being the goal of any pediatric orthopedist. The main problem of achieving this objective is the lack of equipment of the pediatric orthopedic services, a problem that still did not meet with its solution.

Pathologic fracture in solitary bone cyst is usually treated non-operatively; proximal femoral impairment is an exception for this rule.
